# A “Bone Marrow Score” for Predicting Hematological Disease in Immunocompetent Patients With Fevers of Unknown Origin: Erratum

**DOI:** 10.1097/01.md.0000494758.65002.83

**Published:** 2016-08-26

In the article “A “Bone Marrow Score” for Predicting Hematological Disease in Immunocompetent Patients With Fevers of Unknown Origin”,^1^ which appeared in Volume 93, Issue 27 of *Medicine*, a table citation in the Predictive Values of BM Scores section was given incorrectly in the original article. The article has since been corrected online.

## References

[1] Wang H-Y Yang C-F Chiou T-J A “bone marrow score” for predicting hematological disease in immunocompetent patients with fevers of unknown origin. *Medicine*. 2016 93:e243. 25501092 10.1097/MD.0000000000000243 PMC4602808

